# Serological profiling of the EBV immune response in Chronic Fatigue Syndrome using a peptide microarray

**DOI:** 10.1371/journal.pone.0179124

**Published:** 2017-06-12

**Authors:** Madlen Loebel, Maren Eckey, Franziska Sotzny, Elisabeth Hahn, Sandra Bauer, Patricia Grabowski, Johannes Zerweck, Pavlo Holenya, Leif G. Hanitsch, Kirsten Wittke, Peter Borchmann, Jens-Ulrich Rüffer, Falk Hiepe, Klemens Ruprecht, Uta Behrends, Carola Meindl, Hans-Dieter Volk, Ulf Reimer, Carmen Scheibenbogen

**Affiliations:** 1 Institute of Medical Immunology, Charité University Medicine Berlin, Campus Virchow, Berlin, Germany; 2 JPT Peptide Technologies GmbH, Berlin, Germany; 3 Department I of Internal Medicine, University Hospital of Cologne, Cologne, Germany and German Hodgkin Study Group (GHSG), Cologne, Germany; 4 German Fatigue Society e.V. (DFaG), Cologne, Germany; 5 Department of Rheumatology and Clinical Immunology, Charité University Medicine Berlin, Berlin, Germany; 6 Department of Neurology and Clinical and Experimental Multiple Sclerosis Research Center, Charité University Medicine Berlin, Berlin, Germany; 7 Children’s Hospital Schwabing, Technische Universität München (TUM), Munich, Germany; 8 Department of Gene Vectors, Helmholtz Center Munich (HMGU), Munich, Germany; 9 German Center for Infection Research (DZIF), Partner Site Munich, Munich, Germany; 10 Berlin-Brandenburg Center for Regenerative Therapies (BCRT), Charité University Medicine Berlin, Berlin, Germany; University of North Carolina at Chapel Hill, UNITED STATES

## Abstract

**Background:**

Epstein-Barr-Virus (EBV) plays an important role as trigger or cofactor for various autoimmune diseases. In a subset of patients with Chronic Fatigue Syndrome (CFS) disease starts with infectious mononucleosis as late primary EBV-infection, whereby altered levels of EBV-specific antibodies can be observed in another subset of patients.

**Methods:**

We performed a comprehensive mapping of the IgG response against EBV comparing 50 healthy controls with 92 CFS patients using a microarray platform. Patients with multiple sclerosis (MS), systemic lupus erythematosus (SLE) and cancer-related fatigue served as controls. 3054 overlapping peptides were synthesised as 15-mers from 14 different EBV proteins. Array data was validated by ELISA for selected peptides. Prevalence of EBV serotypes was determined by qPCR from throat washing samples.

**Results:**

EBV type 1 infections were found in patients and controls. EBV seroarray profiles between healthy controls and CFS were less divergent than that observed for MS or SLE. We found significantly enhanced IgG responses to several EBNA-6 peptides containing a repeat sequence in CFS patients compared to controls. EBNA-6 peptide IgG responses correlated well with EBNA-6 protein responses. The EBNA-6 repeat region showed sequence homologies to various human proteins.

**Conclusion:**

Patients with CFS had a quite similar EBV IgG antibody response pattern as healthy controls. Enhanced IgG reactivity against an EBNA-6 repeat sequence and against EBNA-6 protein is found in CFS patients. Homologous sequences of various human proteins with this EBNA-6 repeat sequence might be potential targets for antigenic mimicry.

## Introduction

Epstein-Barr Virus (EBV) infection plays a critical role in various autoimmune diseases such as Multiple Sclerosis (MS), Systemic Lupus Erythematosus (SLE), Rheumatoid Arthritis (RA) or Sjögren's syndrome [1–6]. There is a link between infectious mononucleosis and an increased risk for MS and seroprevalence of EBV is close to 100% in MS [7–9]. Several studies show homologies of EBV sequences with human autoantigens such as myelin basic protein for MS [10, 11] and smith antigen for SLE [12].

With an estimated prevalence of 0.3% CFS affects more people than MS and SLE [13]. CFS is a multisystem disorder characterized by severe fatigue with the hallmark symptom of exertion intolerance and post-exertional delay to recover, and cognitive dysfunctions [14, 15]. CFS is probably a heterogeneous disease, but currently no diagnostic biomarkers are available [16–20]. Some studies describe immune dysregulation and activation in CFS patients [21–23]. Numerous studies have tried to find evidence for an association of CFS with EBV. In a subset of patients, CFS begins with infectious mononucleosis and evidence for a potential role of EBV in CFS comes from many studies. In 1984 DuBois first described patients with the mononucleosis syndrome suffering from long-lasting fatigue and serological evidence of EBV reactivation [24] followed by a number of studies describing patients with CFS with serological evidence of chronic active EBV infection [25]. A first placebo-controlled trial with acyclovir in CFS patients with serological active EBV infection performed by Straus *et al*. showed no efficacy [26] while trials with valacyclovir and valganciclovir showed moderate improvement in some patients [27, 28]. Enhanced EBV-specific antibodies against VCA, early antigen and EBV DNAse as well as persistent IgM antibodies were described in numerous studies [29–37] but findings were not consistent [38–40].

In a previous study, we found an elevated IgM response against the late VCA antigen but a lack of antibodies and memory B cells against EBNA-1 in another subset of EBV-positive CFS patients [41]. A diminished EBNA-1 IgG production was also reported in severe infectious mononucleosis and chronic active EBV infection [42–44]. In the present study, we therefore wanted to comprehensively analyse the antibody response against all major EBV proteins in a large cohort of CFS patients. Further, we wanted to answer the question if reactivation of EBV occurs more frequently in CFS patients.

The EBV DNA genome is large and contains over 100 protein-coding genes [45, 46]. In primary infection, EBV undergoes a short period of lytic replication in oral and nasal epithelium [47–49]. The orally transmitted EBV initially targets the mucosal epithelium and remains in a life-long latency in memory B cells [50–52]. In healthy subjects the EBV genome in B cells usually remains latent in the so-called latency phase 0 which maintains the genome in a quiescent state [53–56]. This latency is controlled by NK- and T-cell responses. Frequent replication occurs in epithelial cells of the pharynx. Latency I is characterized by the expression of EBNA-1, latency II by latent membrane proteins (LMP)-1 and LMP-2, and latency III by EBNAs 2–6 [57, 58]. During lytic reactivation the EBV immediate-early genes BZLF-1 and BRLF-1 are expressed. These genes activate viral and cellular promoters that induce early, lytic and late viral gene expression and high amplification of the EBV genome [59]. There are EBV type-specific sequence variations in the EBNA genes that are used to characterize the two EBV major subtypes I or II [44, 60]. The EBV types differ in their capacity to immortalize human B cells with type II EBV as the less efficient strain [61].

In this study, we performed a mapping of the IgG response against 14 EBV proteins by seroarray using overlapping peptide pools in CFS patients and healthy controls. The HHV4 proteome consists of close to 90 proteins of which 8 have been functionally classified as capsid, 14 as membrane protein, 6 as nucleotide metabolism, 9 as latency, 5 as packaging, 7 as replication, 11 as transcription factors, transactivators or involved in signaling and 11 as tegument. We selected one protein from each of these classes except for the latency class where we selected 5 and replication where three were selected. Reactivity against these EBV proteins observed in CFS and healthy controls in our study was rather similar with an enhanced reactivity in CFS patients against a repeat region in EBNA-6.

## Materials and methods

### Study population

CFS patients were diagnosed at the Charité outpatient clinic for immunodeficiencies at the Institute of Medical Immunology at the Charité Universitätsmedizin Berlin between 2011 and 2015. Patients with MS were recruited at the Department of Neurology and patients with SLE at the Department of Rheumatology at the Charité Universitätsmedizin Berlin [8]. Samples of 50 patients with cancer-related fatigue and 50 without fatigue following chemotherapy for Hodgkin’s lymphoma were provided by the University Hospital of Cologne and the German Hodgkin Study Group (GHSG). Diagnosis of CFS was based on Canadian Criteria [62] and exclusion of other medical or neurological diseases which may cause fatigue. Patients with systemic steroid or immunosuppressant therapy or a diagnosis of primary immunodeficiency were excluded from this study. Around 80% of our patients had an onset of disease after acute infection. Baseline demographic characteristics of the patients are shown in Table 1. Controls were recruited from staff and did not suffer from fatigue. However, neither clinical nor laboratory assessment was performed for controls. The study was approved by the Ethics Committee of Charité Universitätsmedizin Berlin in accordance with the 1964 Declaration of Helsinki and its later amendments and patients gave written informed consent.

**Table 1:** Demographic characteristics.

Variable	CFS Peptide Array (n = 92)	CFS Multiwell Array (n = 328)	CFS ELISA (n = 162)	Controls (n = 115)
Age (years)	43 ± 12	43 ± 11	43 ± 11	36 ± 10
m/f (%)	50/ 50	39/ 61	42/ 58	42/ 58
Bell score	30 ± 10	30 ± 10	30 ± 10	n.a.

n.a. not applicable

### Blood samples

Serum obtained from patients and healthy persons was stored in aliquots at -80°C.

### Peptide seroarray

IgG antibody responses to EBV peptides were determined by peptide microarrays as described before [8]. In brief, peptides were synthesized using SPOT synthesis and immobilized onto glass slides (JPT Peptide Technologies, Berlin). Serum samples of healthy donor controls and patients were incubated for 1h and specific antibodies were detected by fluorescently labelled anti-IgG antibodies. Signals were further processed for data analysis and statistical evaluation. Data are reported as arbitrary fluorescence units. Based on our previous study [8] in which we had included two EBV seronegative healthy controls (EBV-VCA- and EBNA-IgG negative) revealing no or only little background reactivity, responder samples were defined as a signal intensity of > 5,000 Units to exclude background signals.

The peptide library contained 3054 peptides covering the full-length EBV proteins BALF-2, BALF-5, BFRF-3 (VP26), BLLF-1, BLLF-3, BLRF-2, BMRF-1, BZLF-1, EBNA-1, EBNA-3, EBNA-4, EBNA-6, LMP-1 and LMP-2 of different EBV strains. Additionally, a relatively conserved region from the N-terminus of VP1 (AA 42–75) of different Coxsackieviruses from the genus Enteroviridae which is described as highly reactive with patient sera was covered by 164 overlapping peptides [63]. The library was printed onto functionalized glass slides in triplicates. The proteins were represented as peptides consisting of 15 amino acids (15-mer) which exhibited optimal overlap of 11 amino acids in most cases to cover the EBV sequence diversity including but not limited to strains of type I (B95.8) and type II (AG876) at minimum peptide numbers (S1 and S2 Files).

### Multiwell assay

IgG antibody responses against a selection of 128 EBV peptides were determined as described elsewhere [64]. In brief, sera were incubated in parallel on multiple identical mini-arrays containing identical copies of triplicates of the peptide library combined on one microarray slide where four slides yielded an incubation frame possessing the dimensions of a 96-well microtiter plate and enabling downstream procedure identical to ELISA (S1 File). The selection of the peptides was based firstly on the p-value for the comparison of the healthy and the CFS patients, respectively. Secondly, peptides with high variances in the signals and negative controls were added.

### ELISA

Selected peptides were synthesized containing an additional C-terminal glycine, a spacer molecule and biotin (JPT Peptide Technologies). 100 nM of biotinylated peptide was coated on a 96 well Streptavidin-plate (Nunc Thermo Scientific) for 1 h at RT. After blocking for 1 h at 30°C serum samples were diluted 1:1,000 and 1:10,000 and incubated for 1 h at 30°C. Secondary antibody (anti-human IgG-HRP (goat), Invitrogen) was diluted 1:4,000 and incubated for 1 h at 30°C, then TMB substrate was added (Sigma Aldrich). Plates were measured at Tecan GENios at 620 nm (S1 File).

### Detection of EBNA-6 protein

EBNA-6 was expressed in HEK293T cells as C-terminally hexahistidine-tagged full-length protein and purified from cell lysates using Nickel-NTA beads (Qiagen). Approximately 50 ng of purified EBNA-6 were spotted onto nitrocellulose membranes (Millipore) and allowed to dry. Subsequently, membranes were blocked with 5% skim milk powder in PBS for 1 h before overnight incubation with patient sera diluted 1:1,000 in 3% skim milk/PBS. Next day, the membranes were incubated for 1 h with a 1:10,000 dilution of IRDye 800CW goat anti-human IgG (Rockland Immunochemicals) in TBS + 0.05% Tween-20 (TBST) and then washed 4 x 5 min in TBST. IR detection was performed with an Odyssey Infrared Imaging System (LI-COR Biosciences) and quantified with the analysis software provided.

### EBV load and calculation of EBER copies

Detection of EBV viral load was done by a quantitative real-time PCR analysis (qPCR) for EBV EBER-1 (Table 2). The DNA samples from throat washing samples were obtained by gargling with 10 ml of water and washed twice with PBS. The pellet was resuspended in 200 μl of AL-Lysis buffer (QIAamp DNA Blood mini Kit from QIAGEN) for DNA preparation according to manufacturer’s instructions. 100 ng DNA template per reaction was added to a final volume of 25 μl. A dilution series of Namalwa DNA, a positive EBV cell line with known EBER copies per specific amount of Namalwa DNA, was used as positive control and to calculate the EBER copies per μg DNA/cDNA of each analysed sample. Results over 35 EBER copies per template DNA or cDNA were regarded as positive. Duplicate measurements of each sample were performed on a 7500 Real Time PCR System (Applied Biosystems Life Technologies). The amplification was performed at 42 cycles with 2 min 50°C, 10 min 95°C and 15 sec 95°C and finally 1 min at 60°C.

**Table 2:** Primer and probe for EBV load and type PCR.

**Primer/ probe**	**Sequence**	**c [nM]**
**EBER forward**	5’-TCC CGG GTA CAA GTC CCG-3‘	108
**EBER reverse**	5‘-TGA CCG AAG ACG GCA GAA AG-3‘	108
**EBER probe**	FAM-5’-TGG TGA GGA CGG TGT CTG TGG TTG TGT T-3‘-TAMRA	200
**EBNA-6 forward**	5’-AGA AGG GGA GCG TGT GTT GT-3’	300
**EBNA-6 reverse**	5’-GGC TCG TTT TTG ACG TCG GC-3’	100

### Detection of EBV type I and II

PCR was performed using primer spanning the EBNA-6 (EBNA-3C) gene (Table 2) as previously described [44]. The primer flank regions in the EBNA-6 gene of type-specific variations resulting in different product fragments with a size of 153 bp for EBV type I and 246 bp for EBV type II. 100 ng DNA template of the throat washing samples were used per reaction. The amplified products were investigated by gel electrophoreses analysis in a 2% agarose gel and visualized by ethidium bromide.

### Statistical analysis

Statistical data analyses were done using R (www.r-project.org) and the software GraphPad Prism 6.0. Nonparametric statistical methods were used. Continuous variables were expressed as median and interquartile range (IQR). Univariate comparisons of two independent groups were done using the Mann-Whitney-U test. Contingency analysis was done by Fisher’s exact test. Correlation analysis was performed by nonparametric Spearman coefficient r. A two-tailed p-value of <0.05 was considered statistically significant.

## Results

### Antibody reactivity against EBV epitopes

**Fig 1:**
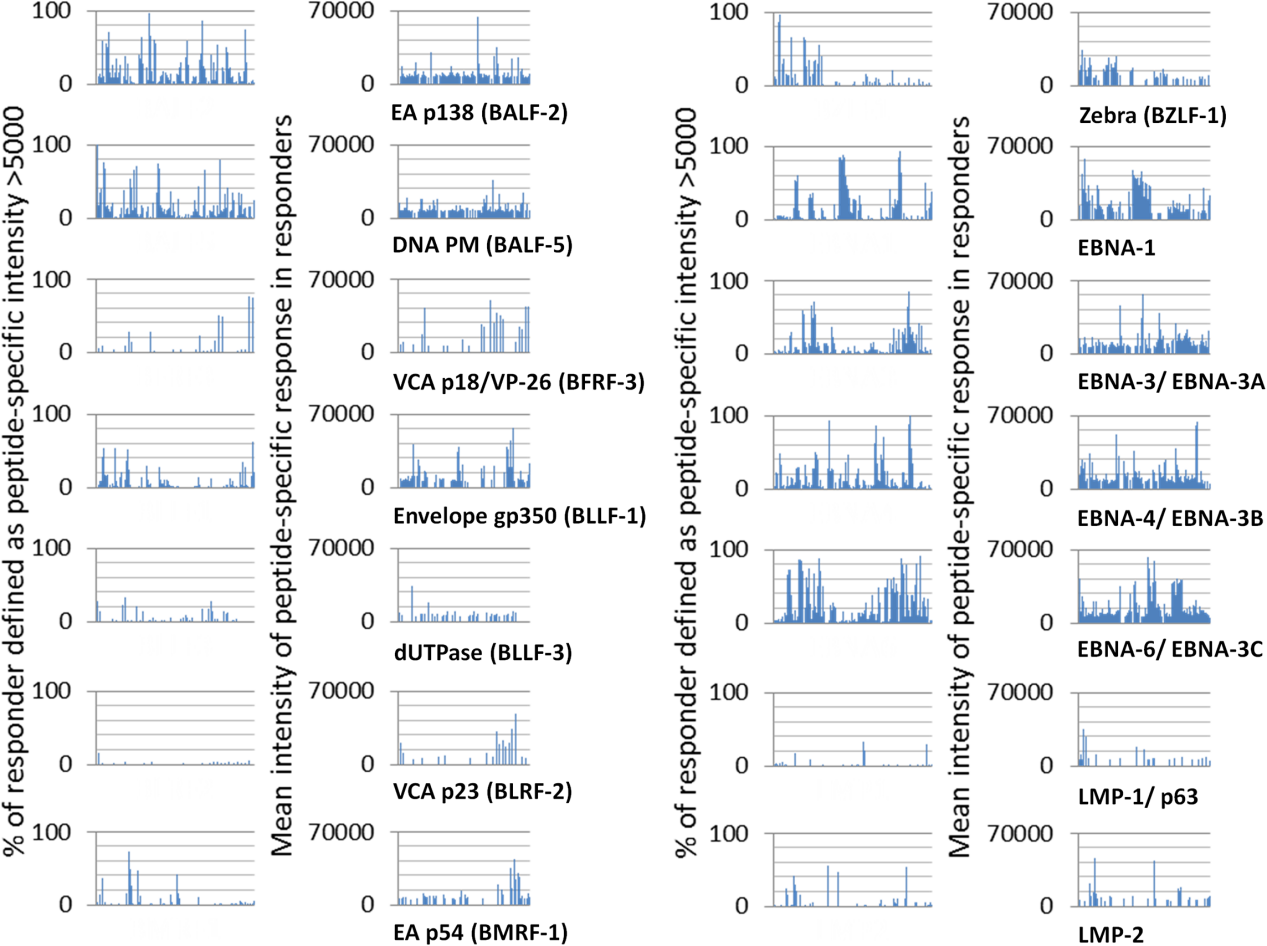
Seroarray analysis of IgG antibody reactivity against 15-mer EBV peptides which cover 14 EBV proteins with sequences of EBV type I (B-95.8) in 50 healthy donors. IgG responses of each sample against each peptide are depicted. Responses are defined as a signal intensity above 5,000. In the left graph the % of samples with a response against each single peptide of the indicated proteins are shown. The right graph shows the mean intensity of these IgG responses (defined as a signal intensity above 5,000) against the single peptides. EA- early antigen; PM- polymerase; VCA- viral capsid antigen; gp- glycoprotein.

Serum IgG responses of 50 healthy controls, 92 CFS, 30 MS, 30 SLE, and 100 Hodgkin’s lymphoma (HD) patients against single overlapping peptides designed as 15-mers of major EBV proteins were comparatively analysed by a seroarray. EBV peptides span different serotypes of the selected proteins BALF-2, BALF-5, BFRF-3, BLLF-1, BLLF-3, BLRF-2, BMRF-1, BZLF-1, EBNA-1, EBNA-3, EBNA-4, EBNA-6, LMP-1 and LMP-2. The IgG reactivity pattern against each peptide of the respective proteins of the 50 healthy controls is shown in Fig 1. Responder samples have a minimum signal intensity of 5,000 Units to exclude threshold signals or noise. The percentage of responders against the single peptides of the indicated proteins is shown in the left graph, and the mean intensity of these IgG responses is shown on the right. We found the most frequent IgG response in controls against peptides of the EBNA proteins. For several EBNA-1, -4 and -6 peptides an antibody response was observed in more than 90% of the 50 healthy controls. In addition, several peptides of early antigens BALF-2 (p138) and BMRF-1 (p54), DNA polymerase BALF-5, and BZLF-1 (Zebra), necessary for EBV replication, were recognized by the majority of healthy controls. In contrast, few peptides from latency phase II LMP-1 and LMP-2, envelope glycoprotein BLLF1 (gp350), dUTPase (BLLF3), viral capsid antigen BLRF-2 (p23) and from the C-terminal region of the viral capsid antigen BFRF3 (p18/VP-26) were recognized by most controls.

**Fig 2:**
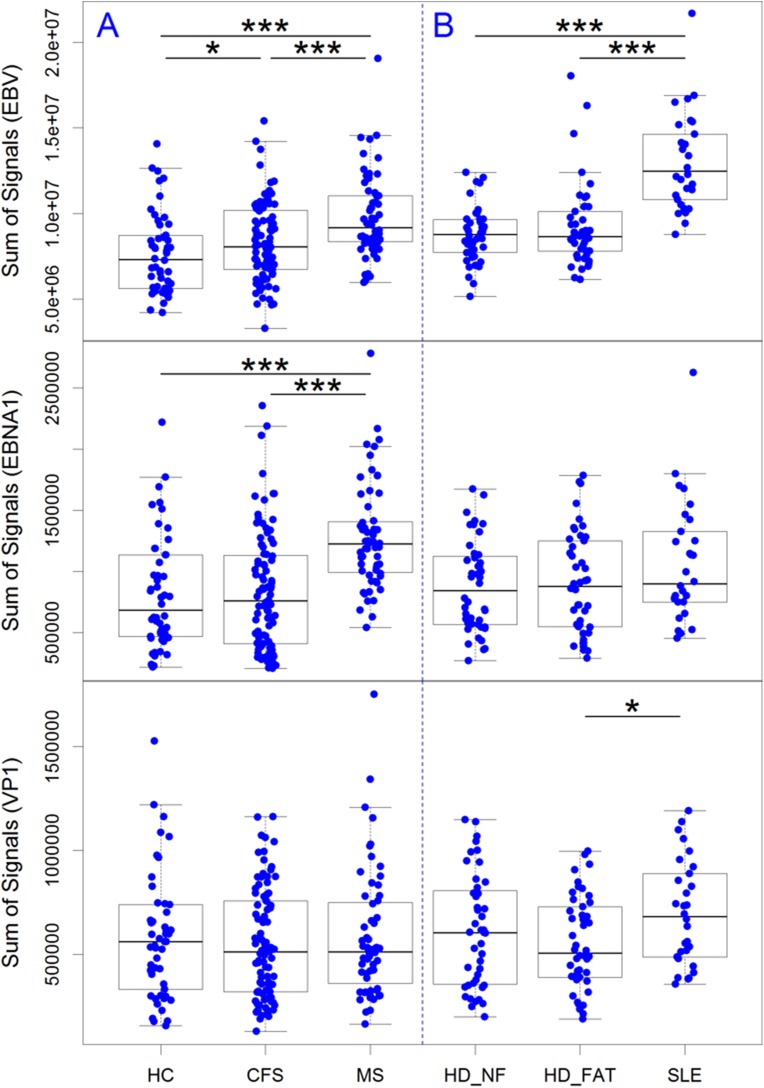
Total IgG responses against EBV, EBNA-1 and enterovirus peptides in the different cohorts. A) The signal intensity of IgG responses against all EBV peptides (upper row), EBNA-1 peptides (middle row) and enterovirus (lower row) in CFS, healthy controls and MS patients. B) Comparison of patients with SLE and Hodgkin’s lymphoma in disease remission with cancer-related fatigue (HD_NF) or without fatigue (HD_FAT) analysed in another seroarray screening experiment. Statistical analysis by Wilcoxon rank sum test with * p<0.05, *** p <0.001.

To illustrate the total IgG responses against all EBV peptides in the different cohorts we calculated a sum of all signal intensities of the IgG responses against each single peptides. As shown in the upper panel of Fig 2A the signal intensity against all EBV peptides was higher in the CFS cohort compared to the healthy controls (p<0.05) and highest in the MS cohort (p = 3.2*10^−6^ against healthy controls). Significant different IgG responses were observed against less than 3% of EBV peptides in CFS compared to healthy controls. The signal difference against the EBNA-1 peptides was higher in MS against healthy (p = 2.8*10^−7^) but not CFS against healthy (middle panel), whereas no difference was found among the cohorts against enterovirus peptides (lower panel). Patients with SLE and patients with HD in disease remission with cancer-related fatigue (HD_NF) or without fatigue (HD_FAT) were analysed in another seroarray experiment (Fig 2B). The signal intensity against all EBV peptides was similar for fatigued compared to non-fatigued Hodgkin patients (upper panel). SLE patients had significantly higher signal intensity against all EBV peptides compared to the cancer patients (p = 3*10^−11^). No or little signal difference was found for the EBNA-1 peptides between the 3 groups (middle panel) while patients with SLE had a higher IgG response to enterovirus peptides (lower panel).

### Enhanced antibody reactivity against an EBNA-6 repeat region in CFS patients

**Fig 3:**
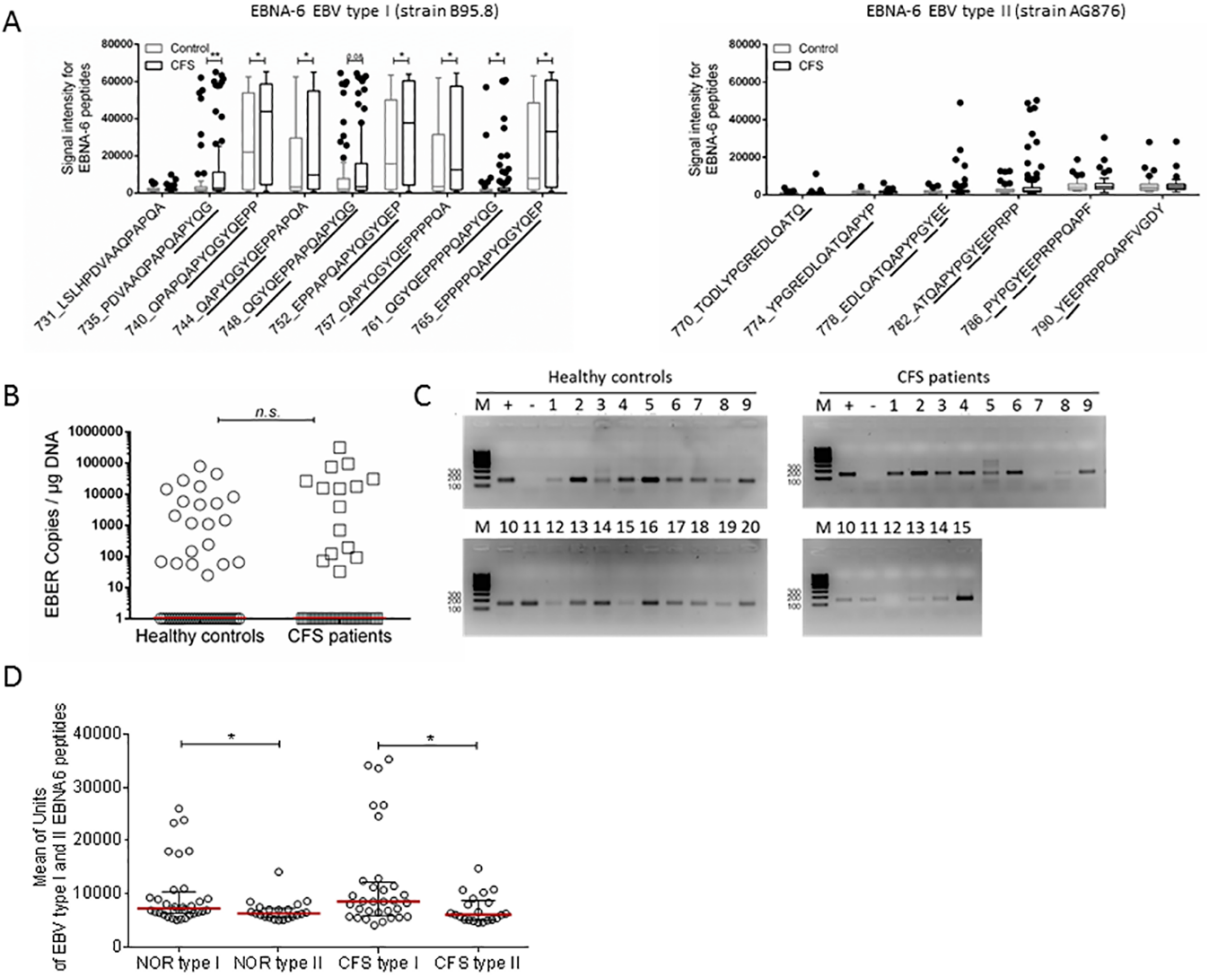
Reactivity against EBV type I EBNA-6. A) Seroarray data showing signal intensities for healthy controls (n = 50) and CFS patients (n = 92) against peptides from repeat region of EBNA-6_731–780 from strain B95.8 of type I EBV (left) and EBNA-6_770–805 from strain AG876 of type II EBV (right) containing the repeat sequence QAPYQGYQE. B) EBER copies were determined by qPCR of EBER-1 in throat washing samples of 50 healthy controls and CFS patients. C) EBER positive samples were tested for EBNA-6 DNA with primers discriminating EBV type I (B95.8) and II (AG876) by PCR. Gel electrophoresis shows 153 bp products corresponding to type I. Product of type II has an estimated size of 246 bp. As positive control (+) EBV type I cell line Namalwa DNA, as negative control (-) water was used instead of template DNA. D) Unique EBNA-6 peptides for EBV serotypes I (B95.8) and II (AG876) were compared for specific IgG response in 50 controls (NOR) and 92 CFS patients (CFS) by seroarray. Shown are EBNA-6 peptides that induced an IgG response of >5000 U in healthy controls (see S2 File). M—DNA ladder. Statistical analysis by Mann-Whitney-U test with n.s.—not significant, * p<0.05, ** p<0.01.

Enhanced reactivity in CFS patients was observed against several peptides from the EBNA-6 aa 744–778 region containing a repeat sequence. All overlapping peptides containing the conserved sequence QAPYQGYQE of type I EBV peptides of EBNA-6 were recognized with a higher intensity by CFS patients compared to healthy controls in the seroarray (Fig 3A, left). The peptides aa 735, 738, and 761 containing a truncated repeat sequence were recognized with lower signal intensity. Further, only a small subset of patients and no controls showed an enhanced IgG response against peptides derived from type II EBV peptides of an EBNA-6 region homologue to the repeat region found in type I (Fig 3A, right). The repeat sequence QAPY**P**GY**E**E varied in two aa.

### Prevalence of type I EBV in CFS patients

Throat washing samples as a source of frequent EBV reactivation were collected from 50 CFS patients and 50 healthy donors. The samples were measured by qPCR for EBV viral load by calculating copies of EBER per μg template DNA. EBER copies were detected in 42% (n = 21) of healthy donors and 30% (n = 15) of CFS patients (Fig 3B) revealing no significant difference in frequency (p = 0.37). Samples of EBER positive patients and controls were further analysed for the prevalence of either EBV type I or II. Here a PCR was established using primer spanning the EBNA-6 gene, as previously described [44]. The primers flank regions in the EBNA-6 gene resulting in type-specific products of different sizes (Fig 3C). The resulting fragments after gel electrophoresis show products with a size corresponding to 153 bp for EBV type I (B95.8) in all samples of both patients and controls. No signals for EBV type II (AG876) expected at 246 bp were observed. No clear product was detected for two CFS patients due to low DNA concentrations and the lowest EBER copy number in the PCR analysis. Data from the seroarray are in accordance to our finding of the prevalence of EBV type I in controls and patients. We detected significantly enhanced IgG responses in all controls and patients against EBNA-6 peptides specific for type I compared to type II (Fig 3D). Here, peptides are shown for which an IgG response of >5000 U was observed in healthy controls.

### Evidence for enhanced IgG-response against EBNA-6 protein in CFS and potential mimicry with human lactoperoxidase

**Fig 4:**
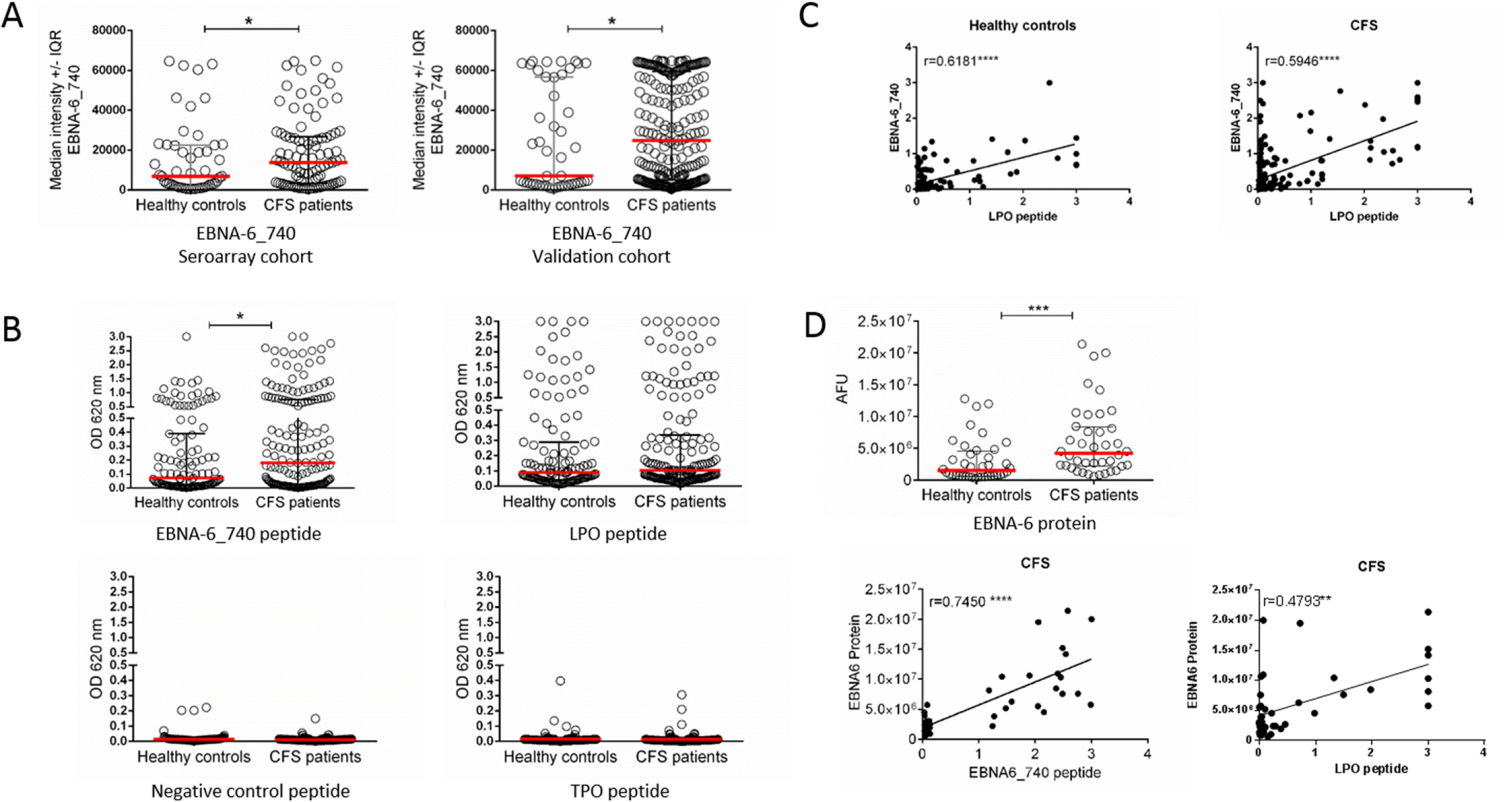
Reactivity against EBNA-6, LPO and TPO in CFS patients. A) Median intensity of IgG response against EBNA-6 peptide 740 in seroarray cohort (CFS, n = 93 and healthy, n = 50) and a subsequent validation cohort (CFS, n = 227 and healthy, n = 47) was analysed by multiwell assay. B) Optical density (OD) and Interquartile Range (IQR) of IgG response against EBNA6_740, a negative control peptide, LPO, and TPO in healthy controls (n = 115) and CFS patients (n = 162) by peptide ELISA. C) Correlation of ELISA OD values of EBNA6_740 and LPO for all patients and controls. D) IgG responses against EBNA-6 protein in in healthy controls (n = 40) and CFS patients (n = 40) detected by Odyssey Infrared Imaging System as arbitrary fluorescence units (AFU), and correlation of EBNA-6 protein and EBNA-6 peptide as well as LPO peptide IgG in CFS patients. Statistical analysis by two-tailed Mann-Whitney-U test with * p<0.05, ** p<0.01, *** p <0.001, **** p<0.0001, r-spearman coefficient.

The enhanced IgG response against the EBNA-6 repeat region of type I EBNA-6 could be confirmed in the multiwell assay in which 128 selected peptides based on the results of the seroarray were tested. In both the cohort analysed by seroarray (CFS, n = 93 and healthy, n = 50) and a subsequent validation cohort (CFS, n = 227 and healthy, n = 47) (Fig 4A) we found IgG responses against the EBNA-6 peptide at higher intensities in patients compared to controls. Further, the antibody response against EBNA-6_740 containing the repeat region was assessed by a peptide ELISA (Fig 4B) confirming a significantly enhanced antibody response in 162 CFS patients compared to 115 healthy donors (p = 0.02). IgG responses >95%ile of healthy controls (OD of 0.882) were found in 14.8% of CFS patients (p = 0.02). A peptide which was not recognized in the seroarray was used as negative control.

The core sequence of the repeat region of EBNA-6 (QAPYQGYQE) was studied for homologue sequences in human proteins using BLAST the Basic Local Alignment Search (database: UniProtKBSwiss-Prot, sorted by MaxScore) revealing a 7 amino acids homologous region comprising 6 identical amino acids in the human LPO protein sequence (Table 3). Further proteins showing at least 4 amino acid identity to the EBNA-6 repeat region were thyroid peroxidase (TPO), phosphofructokinase (PFK) and the ornithine carbamoyltransferase (OTC). All 3 are of interest, because their function may be linked to CFS pathogenesis [65, 66]. In a next step, 15-mer peptides covering the homologue sequence of LPO or TPO were synthesized and the antibody response was tested in the same 162 CFS patients and 115 controls by peptide ELISA. 30 of 58 CFS patients with elevated EBNA6_740 IgG (defined as above the IQR of healthy controls) had elevated LPO IgG as well. We observed a significant correlation of reactivity against EBNA-6 peptide and LPO peptide in patients (Fig 4C). However, healthy controls showed a similar cross-reactivity and there was no significant difference in LPO peptide reactivity between patients and controls in the ELISA (Fig 4B). No elevated IgG responses against the TPO peptide were observed in 162 CFS patients and 115 healthy controls (Fig 4B).

**Table 3:** Homologue sequences of EBNA6_740 to human proteins.

**Protein**	**Sequence**	**Sequence**
**EBNA-6**	UniProtKB-P03204 (EBNA6_EBVB9) aa 740–754	QPAP**QAPYQGYQE**PP
**LPO**	UniProtKB-P22079-1 (PERL_HUMAN) aa 438–452	MQKWIP**PYQGY**S**E**SV
**TPO**	UniProtKB-P07202-1 (PERT_HUMAN) aa 463–477	FQQYVG**PY**E**GY**DSTA
**OTC**	UniProtKB-P00480 (OTC_HUMAN) aa 275–288	KKRL**QA**-F**QGYQ**VTM
**PFKP**	UniProtKB-Q01813-1 (PFKAP_HUMAN) aa 61–65	GAKVYFI**Y**E**GYQ**GMV

Sequence homology search was performed using BLAST [database: UniProtKBSwiss-Prot]. Homologous sequences to EBNA-6 were underlined and identical amino acids were printed in bold letters. Repeat sequence in EBNA-6 was highlighted as well.

EBNA: Epstein-Barr nuclear antigen, LPO: lactoperoxidase, TPO: thyroid peroxidase, OTC: ornithine carbamoyltransferase, PFKP: platelet type 6-phosphofructokinase.

We next analysed IgG responses against EBNA-6 protein in a subset of the patients analysed for EBNA-6 peptide responses. We observed significantly enhanced IgG responses against the EBNA-6 protein in CFS patients versus controls as well as a good correlation between recombinant EBNA-6 protein and EBNA-6 peptide IgG (Fig 4C).

Further IgG responses against recombinant LPO protein as well against PFK-P and OTC proteins were analysed in in a subset of the patients and healthy donors analysed for EBNA-6 peptide responses by ELISA. We observed neither clearly enhanced IgG responses nor differences between CFS and healthy controls against these 3 proteins (data not shown).

## Discussion

In this study we performed a comprehensive analysis of IgG responses against a peptide library of the major EBV proteins. In healthy controls we observed the strongest and broadest antibody responses against the latency proteins EBNA-1, -3, -4, and -6, and the lytic proteins BALF2, BALF5, and BZLF1. This finding is in accordance with studies analysing the IgG response against EBV proteins in which strong IgG responses against both EBNA proteins and BZLF1 are found and used for serodiagnosis [67–73]. The strong enhanced IgG response against various EBV proteins in MS and SLE shown in previous studies was confirmed in this and for MS in our prior study [8]. In contrast, although total IgG response against EBV peptides was higher in CFS the pattern of IgG responses was more similar to healthy controls. Further, we had performed a subgroup analysis in patients with Hodgkin lymphoma showing no difference in IgG EBV response pattern in patients with and without fatigue.

Interestingly, we observed a significantly enhanced IgG response against EBNA-6 peptides spanning a repeat region in CFS patients compared to healthy controls. A 21 aa long peptide comprising the repeat region of EBNA-6 was already described by Rajnavölgyi *et al*. as a HLA-DR restricted T cell epitope [74]. Further we could show a close correlation between IgG responses against EBNA-6 peptide and a recombinant EBNA-6 protein suggesting that this peptide is recognized in the protein and is an immunodominant epitope. If the enhanced IgG response against EBNA-6 may be of diagnostic relevance in a subgroup of patients needs to be analysed in a longitudinal study. Cross-reactivity of EBV-specific IgG with human antigens due to antigenic mimicry is known to trigger pathogenic immune responses in SLE and MS [75–77]. We thus performed a sequence comparison of the EBNA-6 repeat region with human proteins and identified a 7 amino acids homologous sequence in the LPO protein, an enzyme producing oxidants and secreted by mammary, salivary and mucous glands of the bronchia. LPO is involved in immune defence with broad activity against bacteria and viruses [78–80]. Interestingly, we detected a correlation of EBNA-6 peptide and protein IgG with LPO peptide IgG levels. However, we observed no elevated levels of LPO protein IgG in patients with CFS suggesting that the peptide is not recognized in the recombinant protein. Interestingly, human TPO shows a sequence homology with 5 (comprising 4 identical aa) of the 7 amino acids of the EBNA-6 repeat region as well. Autoantibodies against TPO and hashimoto's thyroiditis are detected in 10–20% of patients with CFS and lead to thyroid destruction through antibody dependent cellular cytotoxicity and complement activation [81]. We could, however, not observe elevated IgG responses against the TPO peptide in our study. Two other proteins showing homology to the EBNA-6 repeat region, the enzymes OTC and PFK arouse our interest because of their metabolic function (Table 3). Data from Yamano *et al*. revealed higher ornithine/citrulline ratio in CFS patient which could be explained by reduced OTC activity [66]. The PFK catalyses the rate limiting step of glycolysis and inhibition of the enzyme could play a role in metabolic alterations in CFS [65, 66]. However, as for LPO protein, we observed no reactivity of CFS patients against these two proteins.

There exist two major EBV subtypes type I and type II EBV with sequence variations in several EBV proteins. The prevalence of EBV serotypes varies in different geographic regions [51]. While healthy subjects usually are resistant to coinfection with another subtype, in immunodeficiency infection with two EBV types is frequently found [82, 83]. In our study, there was a significantly lower IgG response against peptides from type II specific regions compared to type I specific regions in both CFS patients and controls. EBV frequently reactivates in the oropharynx and can be detected in throat washing samples in 20–70% in healthy donors [84, 85]. Our data show a similar prevalence of EBV reactivation in 30–40% of samples and the infection with type I EBV in both healthy controls and CFS patients providing no evidence for EBV coinfection or prevalence of another EBV serotype in CFS patients. The prevalence of EBV type I is in accordance with another study showing that it is more frequent in northern countries [51]. The observed antibody responses against peptides derived from sequences of EBV type II strains might be due to cross reactivity.

In conclusion, we could show the suitability of the EBV peptide microarray to analyse the seroresponse against EBV. Further, we could identify multiple peptide epitopes of EBV proteins recognized in healthy controls and patients. Our seroarray data and the similar prevalence of EBV in throat washings in CFS compared to healthy controls, argue against a pathogenic role of EBV reactivation in CFS. The enhanced IgG response against an EBNA-6 repeat sequence may point to a potential antigenic mimicry and requires further studies.

## Supporting information

S1 File Raw data of peptide seroarray, multiwell array and peptide ELISA. (XLSX)

S2 File EBNA-6 type I and type II specific peptide sequences shown in Fig 2D. (DOCX)
