# Supplementary material for: Serological profiling of the EBV immune response in Chronic Fatigue Syndrome using a peptide microarray
Source: PLoS One. 2017 Jun 12;12(6):e0179124. doi: 10.1371/journal.pone.0179124 (PMC5467847; doi:10.1371/journal.pone.0179124)
Supplement: S2 File — (DOCX) [file pone.0179124.s002.docx]

**S2 File. Table.** EBNA6 peptide sequences used in figure 2 D.

| EBNA-6 peptide sequences | |
| --- | --- |
| EBV type I | **EBV type II** |
| 740_QPAPQAPYQGYQEPP | 086_HLLDMEDNVPPWFPP |
| 752_EPPAPQAPYQGYQEP | 094_VPPWFPPHDITPYVA |
| 765_EPPPPQAPYQGYQEP | 098_FPPHDITPYVARNIR |
| 744_QAPYQGYQEPPAPQA | 102_DITPYVARNIRDAAC |
| 770_QAPYQGYQEPPAHGL | 158_GPLVVEGGVGWRHWL |
| 757_QAPYQGYQEPPPPQA | 167_GWRHWLLTSPSRSWS |
| 790_ PGYAGPWTPRSQHPC | 171_WLLTSPSRSWSMGYR |
| 748_ QGYQEPPAPQAPYQG | 175_SPSRSWSMGYRTATL |
| 822_QGPWDPRAPHLPPQW | 226_VWIPPPAGPREQERY |
| 338_VIQNAFRKAQIQGLS | 298_NSSSVNYWFHKTIGN |
| 158_GPLVAEGGVGWRHWL | 597_RPRVMAPPSTGPRVM |
| 167_GWRHWLLTSPSQSWP | 670_GPAPRSFWEMRAGRD |
| 794_GPWTPRSQHPCYRHP | 790_YEEPRPPQAPFVGDY |
| 798_PRSQHPCYRHPWAPW | 794_RPPQAPFVGDYGFVQ |
| 735_PDVAAQPAPQAPYQG | 830_QLPAALDLGPEQPRF |
| 806_RHPWAPWSQDPVHGH | 858_SCPGYAGPWPSRPQH |
| 802_HPCYRHPWAPWSQDP | 882_WPREPRHGHSQGPWK |
| 584_AGPPAAGPPAAGPRI | 886_PRHGHSQGPWKPWSA |
| 171_WLLTSPSQSWPMGYR | 890_HSQGPWKPWSAHLPP |
| 094_VPPWLPPHDITPYTA | 894_PWKPWSAHLPPQWDG |
| 210_FGCQNAARTLNTFSA | 929_PPRLQLSSVPQVLYP |
| 264_IELCGSLHHIWQNLL |  |
| 639_PQSTGRKPQCFWEMR |  |
| 298_NNPAVNYWFHKTIGN |  |
| 565_GPPAAGPPAAGPPAA |  |
| 561_PPAVGPPAAGPPAAG |  |
| 294_MSSCNNPAVNYWFHK |  |
| 334_IKEHVIQNAFRKAQI |  |
| 588_AAGPPAAGPRILAPL |  |
| 412_PAKKPRKLPWPTPKT |  |
| 222_FSATVWTPPHAGPRE |  |
| 786_SSSYPGYAGPWTPRS |  |
